# Confounding

**DOI:** 10.4103/0019-5545.33263

**Published:** 2007

**Authors:** Chittaranjan Andrade

**Affiliations:** Department of Psychopharmacology, National Institute of Mental Health and Neurosciences, Bangalore - 560 029, India

**Keywords:** Bias, confounding, research methods

## Abstract

**Background:**

Confounding is a statistical concept that is important to all researchers.

**Materials and Methods:**

The concept of confounding is explained with the help of an amusing but true example. Simple explanations about and examples of confounding are provided. Methods to deal with confounding are detailed and their applications and disadvantages are examined.

**Conclusions:**

Attention to confounding is necessary at the time of study design as well as during the statistical analysis of data. The failure to identify and control for confounding can result in the faulty interpretation of study outcomes.

## INTRODUCTION

Axial myopia is the commonest form of myopia. It arises out of an excessive growth of the eyeball during childhood and adolescence and results in images being focused in front of rather than upon the retina. Research in species as diverse as chicks and monkeys suggests that postnatal eyeball growth is influenced by a vision-dependent retinal mechanism which utilizes local pathways in the eye; extraocular neural pathways and the brain are little involved.

Simply put, the duration of the daily light period affects eyeball growth. Therefore, might axial myopia result from undue light exposure, such as might occur when lights are left on at night?

## DOES NIGHT LIGHTING INCREASE THE RISK OF MYOPIA?

The issue was investigated by Quinn *et al*[1] in a paper which received much publicity in the mass media. The study sample comprised 479 myopic children at a tertiary referral centre. The sample was aged 2-16 years (median age, 8 years) and was 55% male; children with other ocular disorders or a history of prematurity were excluded. Parents completed a questionnaire that recorded the child's exposure to light at the time of the study as well as during early childhood.

The findings were that exposure to nighttime light before the age of two years was related to the incidence of myopia in a dose-dependent fashion: in children who had slept in the dark, in those who had slept with a night light, and in those who had slept with full room lighting, the incidence of myopia was 9%, 31%, and 48% respectively.

There was no relationship between early childhood nursery lighting and other errors of refraction, nor a relationship between current night light exposure and errors of refraction. Quinn *et al.* concluded that, as the eyelids are not totally opaque to light, nighttime lighting during the first two years of life might stimulate eyeball growth and predispose to the later development of myopia. Quinn *et al* therefore recommended that infants and young children sleep at night without artificial lighting in the bedroom.

## NIGHT LIGHTING IS HARMLESS!

Quinn *et al.* had published their results during May, 1999. Nearly a year later, responses were published from other researchers who had attempted but failed to replicate the findings. In brief, neither Zadnik *et al.*,[2] with a sample size of 1220 children, nor Gwiazda *et al.*,[3] with a sample size of 213 children, found any relationship between the presence or intensity of nighttime lighting before the age of two years and the risk of myopia. However, both groups of researchers did obtain another result: that myopic parents were more likely to leave the lights on at night.

This finding explains the results presented by Quinn *et al*.[1] Myopic parents, more than emetropic parents, may require lights to see well at night; as infants often require nighttime visits, this might explain night lights being left on in the bedrooms of the infants. Genetic factors are known to influence the etiology of myopia; therefore, the association between nighttime lighting during early childhood and the later development of myopia is explained on the basis of a confounding variable: parental myopia.

As a tailpiece to this story, Quinn and his colleagues[4] did not accept this explanation graciously. Perhaps scientists can be as stubborn as politicians who are proven wrong.

## EXPLAINING CONFOUNDING FURTHER

Confounding is said to exist when a third factor, known as the confounding variable, explains the association between two variables. For example, schizophrenia may be more prevalent in lower socioeconomic strata not necessarily because poverty predisposes to the disorder but because schizophrenia compromises social and vocational competence; as a result, affected individuals may drift down the social scale. The compromised social and vocational competence is the confounding variable which at least in part explains the relationship between schizophrenia and lower socioeconomic status.[5]

Likewise, an association between depression and coronary heart disease events could, at least in part, because depressed patients are less likely to exercise and less likely to comply with dietary restrictions and cardiovascular pharmacotherapy regimens.[6] These behaviors comprise the confounding variables.

## PREVENTING OR CONTROLLING FOR CONFOUNDING IN RESEARCH

Confounding can be prevented or corrected in at least four different ways:

1. *By restriction;* for example, if the use of hormone replacement therapy confounds the risk of Alzheimer's disease[7] in a hypothetical study of the efficacy of a vaccine for the condition, the sample for the study can be restricted to women who have not used hormone replacement therapy. Restriction makes it more difficult to obtain a sample of adequate size and limits the generalizability (and hence the external validity) of the study.
2. *By matching;* in the previous example, each woman who had used hormone replacement therapy could be matched with a woman who had never used hormone replacements. Matching may not be easy. For example, age, gender, family history, education, diet, use of hormone replacements, long-term use of anti-inflammatory drugs, and other factors are all confounding variables in any study of the risk of Alzheimer's disease[8,9] and it would be very difficult to effect one-to-one matching of subjects on all these variables. Another problem with matching is that, if subjects are matched on a particular variable, the effect of that variable (on the outcome of the study) cannot be statistically examined.
3. *By stratification;* in the previous example, women with and without a history of hormone replacement therapy could be independently randomized to receive vaccine or placebo; or, the risk of Alzheimer's disease with vaccine versus placebo could be independently studied in women with and without a history of hormone replacement therapy. However, stratification could become problematic if there are multiple confounding variables because multiple layers of stratification would then become necessary.
4. *By using statistical procedures;* an analysis of covariance can be used to control for a single confounding variable and one of several types of regression analysis can be used to simultaneously control for several confounding variables. There are, however, certain assumptions to be fulfilled for such analyses and certain limitations to the results that are obtained.[10]

## CONCLUDING NOTES

Confounding can be addressed only if it is known or suspected. For example, in the night lighting example presented in the opening sections of this article, if Zadnik *et al*[2] and Gwiazda *et al*[3] had not know about the genetics of axial myopia, they would not have examined parental refraction as a confounding variable and would not have refuted the conclusions of Quinn *et al*.[1] As a result, three studies would have wrongly concluded that leaving night lights on during infancy will predispose to axial myopia in later life.

Confounding can be addressed only if the confounding variable has been measured. For example, in a study of whether or not diet modifies the risk of Alzheimer's disease, the confounding effect of hormone replacement therapy can be statistically controlled for only if the use of hormone replacement therapy was recorded at the time of data collection.

Studies often fail to collect data on all sources of confound. Sometimes, this may be because the source of confound is not known. Sometimes, this may be because the source of confound was known but, regrettably, was not measured. In both cases, confounding can never be controlled for and the conclusions of the study can be wrong. The story of nighttime lighting and myopia is a classic case in point.

Investigators who initiate research should therefore strive to record all possible sources of confound in their study. Towards this goal, a close liaison with a statistician and research methodologist is called for, both at the time of study design and at the time of data analysis. A further discussion on sources of bias in research was provided by Grimes and Schulz.[11]
